# Supplementary material for: Changes in Bone Marrow Fatty Acids Early after Ovariectomy-Induced Osteoporosis in Rats and Potential Functions
Source: Metabolites. 2022 Dec 26;13(1):36. doi: 10.3390/metabo13010036 (PMC9863616; doi:10.3390/metabo13010036)
Supplement: Supplementary file 1 [file metabolites-13-00036-s001.zip › metabolites-2070203-supplementary.pdf]

Table S1. VIP values of fatty acids on the 3rd day

Fatty acids	VIP values
Palmitoleate	2.691585693
Myristate	2.075778242
Arachidonate	1.533928522
Docosahexaenoate	0.84502846
Myristoleate	0.820132861
Heptadecanoate	0.776451813
Alpha linolenate	0.752873546
Eicosadienoate	0.745937418
Nervonoate	0.502646536
Stearate	0.469915765
Homogamma Linolenate	0.465295352
Laurate	0.380375104
Gamma linolenate	0.247806882
Palmitate	0.193000772
Arachidate	0.162783227
Erucate	0.139940784
Eicosenoate	0.036941738
Pentadecanoate	0.001188429

Table S2. VIP values of fatty acids on the 14th day

Fatty acids	VIP values
Palmitoleate	1.730463048
Myristate	1.663763778
Alpha linolenate	1.648455561
Gamma linolenate	1.463276233
Pentadecanoate	1.199061055
Stearate	1.16163541
Eicosenoate	1.049586923
Eicosadienoate	0.993133164
Laurate	0.956790419
Heptadecanoate	0.720661481
Arachidonate	0.592671264
Homogamma Linolenate	0.445040643
Erucate	0.440255224
Myristoleate	0.327426894
Palmitate	0.259042422
Nervonoate	0.239338966
Arachidate	0.226210643
Docosahexaenoate	0.207370564

Table S3. Chemical formula of fatty acids

Chemical formula	Fatty acid
C12:0	Laurate
C14:0	Myristate
C14:1	Myristoleate
C15:0	Pentadecanoate
C16:0	Palmitate
C16:1	Palmitoleate
C17:0	Heptadecanoate
C18:0	Stearate
C20:0	Arachidate
C18:3N6	Gamma Linolenate
C20:1	Eicosenoate
C18:3N3	Alpha Linolenate
C20:2	11-14 Eicosadienoate
C20:3N6	Homogamma Linolenate
C22:1N9	Erucate
C20:4N6	Arachidonate
C24:1	Nervonoate
C22:6N3	Docosahexaenoate
